# Case report: From negative to positive: a remarkable journey of ER, PR and HER2 status in a patient with metastatic breast cancer

**DOI:** 10.3389/fonc.2024.1381541

**Published:** 2024-04-26

**Authors:** Jiaqi Huang, Lan Liu, Jianghua Ding

**Affiliations:** ^1^ Department of Oncology, Jiujiang University Affiliated Hospital, Jiujiang, Jiangxi, China; ^2^ Department of Ultrasonic Diagnosis, Jiujiang University Affiliated Hospital, Jiujiang, Jiangxi, China

**Keywords:** breast cancer, ER, PR, HER2, transformation, negative, positive

## Abstract

Breast cancer is the most common malignant tumor in women, posing a serious threat to women’s health. HER2 has been identified as a key oncogene and prognostic factor in breast cancer. Recent studies have reported inconsistencies in ER, PR, and/or HER2 expression between primary breast tumors and metastatic lesions. Rarely is it reported that all three biomarkers experience conversion. In this report, we present the case of a female patient with relapsed and metastatic breast cancer, whose histology transformed from initially triple-negative to Luminal-B type (HER2 positive) (i.e., ER, PR, and HER2 positive). She underwent systematic chemotherapy, targeted therapy, and cranial radiotherapy, which was followed by maintenance treatment with targeted and endocrine therapy. Currently, she has been in nearly complete remission (nCR) for more than 12 months. For recurrent and metastatic breast cancer, it is necessary to perform the second biopsy for metastases, which would contribute to precision treatment and prognosis improvement.

## Introduction

According to the latest National Cancer Report, breast cancer is the most common malignant tumor and the second leading cause of cancer mortality in Chinese females (1). The therapeutic strategies for newly-diagnosed breast cancer include surgery, chemotherapy, radiotherapy, targeted therapy, and endocrine therapy. The choice of treatment depends on the stage of TNM, pathology, molecular types, and physical conditions (2). For relapsed and metastatic breast cancer, the treatment strategy is primarily based on the original pathological and molecular types of the cancer (2). Recently, several retrospective studies have reported inconsistent biomarkers, i.e. estrogen receptor (ER), progesterone receptor (PR), and human epidermal growth factor receptor-2 (HER2), between the primary lesions and metastases of breast cancer (3–6). The majority of the discordance occurred only one or two of ER, PR and HER2 status. It is rarely reported that all three biomarkers simultaneously changed between primary lesion and recurrent & metastases. In this report, we present a case of a female patient with stage IIIA breast cancer who experienced a transition from initial triple-negative to Luminal-B type (HER2 positive) after recurrence and metastasis. As a result, she received anti-HER2 therapy plus chemotherapy and achieved nearly complete remission (nCR).

## Case report

**Figure 1:**
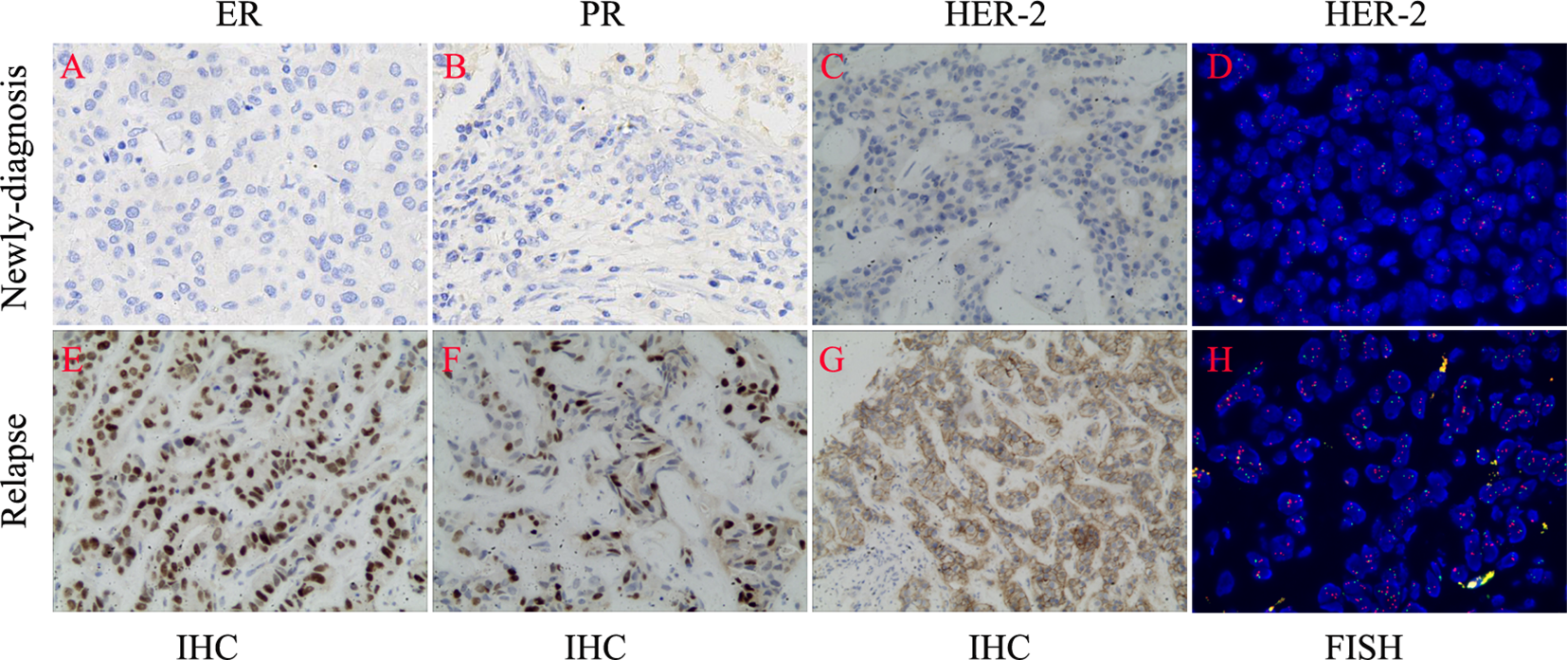
The changes of biomarkers between newly-diagnosed and relapsed biopsies. **(A–D)** Negative expression of ER, PR, and HER-2. **(E–H)** Positive expression of ER, PR, and HER-2.

On March 2, 2018, a 31-year-old woman complained of a palpable mass in the left breast for more than 2 months. The serum levels of CEA and CA153 were 22.5ng/ml and 46.8U/ml, respectively. Then she was diagnosed with left breast cancer and underwent a modified radical resection at Jiujiang University Affiliated Hospital. Postoperative pathology revealed invasive ductal carcinoma of the left breast, non-specific type, with left axillary lymph nodes metastasis (5/23). Immunohistochemical examinations showed ER (-), PR (-), HER2 (-), and Ki-67 (80% +), indicating triple-negative type breast cancer (**Figure 1**). Because the patient possessed a certain degree of medical knowledge, she required further examination of HER2 by fluorescence *in situ* hybridization (FISH). The negative status of HER2 was typically confirmed by FISH.

The stage of TNM was pT2N2aM0, stage IIIA. Due to the high risk of relapse (≥4 axillary lymph node positive), the patient received adjuvant chemotherapy since April 4, 2018. Specifically, the combined regimen consisted of epriubicin and cyclophosphamide (EC) for 4 cycles with 1 cycle every 3 weeks. Then, she was treated with albumin-bound paclitaxel (T) for 4 cycles per 3 weeks cycle. On Oct. 10, adjuvant radiotherapy was administered to the chest wall and regional lymph node drainage field, with 25 daily doses (fractions) of 2.0 Gy to a total dose of 50Gy over 5 weeks. The patient was followed up regularly and examined every 3 months during the first 2 years after surgery, including tumor markers, breast ultrasound, chest and abdominal CT, and brain MRI examinations. Sine the third year after operation, she received regular evaluation every 6 months. The patient was in good condition and no signs of tumor recurrence were found.

**Figure 2:**
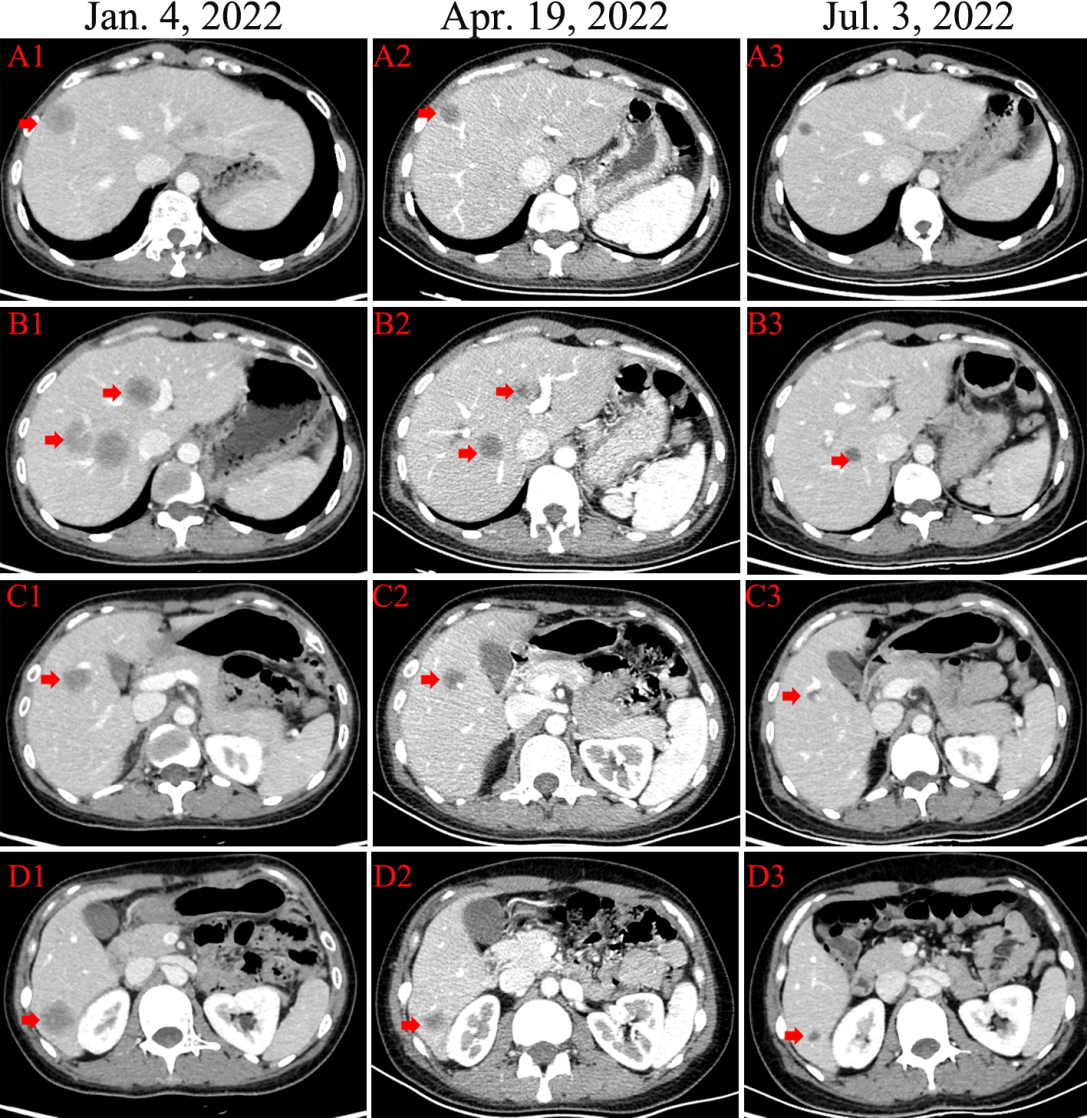
The CT changes of different metastatic lesions from liver **(A–D)**.

In December 2022, the patient complained of right upper abdominal discomfort, loss of appetite and occasional nausea. The patient was examined at our Hospital on December 22, 2022. The CEA level was 32.86ng/ml. The CA153 level was greater than 342.5ng/ml. Abdominal CT indicated multiple metastatic tumors of the liver (**Figure 2**). On January 4, 2023, the brain magnetic resonance imaging (MRI) was performed. It found an abnormal signal in the right frontal nodule, suggesting metastasis. Cervical lymph node ultrasonography showed abnormal structural lymph nodes in the bilateral neck region II and left neck region V. The maximum diameter of lymph nodes was only 1.4 cm. Also, the physical examination showed no significantly palpable superficial lymph nodes. Thus, the patient received percutaneous color ultrasound-guided needle biopsy of liver biopsies on January 4, 2023. Pathological findings supported that the liver metastases were consistent with breast cancer. Immunohistochemistry results showed ER (70% +), PR (40% +), Ki-67 (40% +), as well as the positive expression of HER2 (2+). Furthermore, the positive status of HER2 was confirmed by FISH detection (**Figure 1**). Finally, the patient was diagnosed with Luminal B type (HER2 positive), i.e. ER, PR, and HER2 positive. The patient received stereotactic radiotherapy (SRT) with DT50Gy/10F for the solid brain metastasis on January 8, 2023. Concurrently, the patient received the combined treatment of docetaxel(D), trastuzumab (H), and pertuzumab(P) (D-HP) for 6 cycles, with each cycle lasting 3 weeks, starting from January 11, 2023. The clinical efficacy was evaluated based on the Response Evaluation Criteria for Solid Tumors (RECIST1.1), including complete response (CR) and partial remission (PR). CR indicates that all lesions disappear completely, and all pathological lymph nodes reduce to normal size (short diameter < 1cm), lasting for more than 1 month. PR means that the total maximum diameter of all measurable lesions reduces by over 30%, lasting for more than 1 month. Nearly CR (nCR) refers to that the majority of all measurable lesions disappear, but only a few tiny residual foci remain.

**Figure 3:**
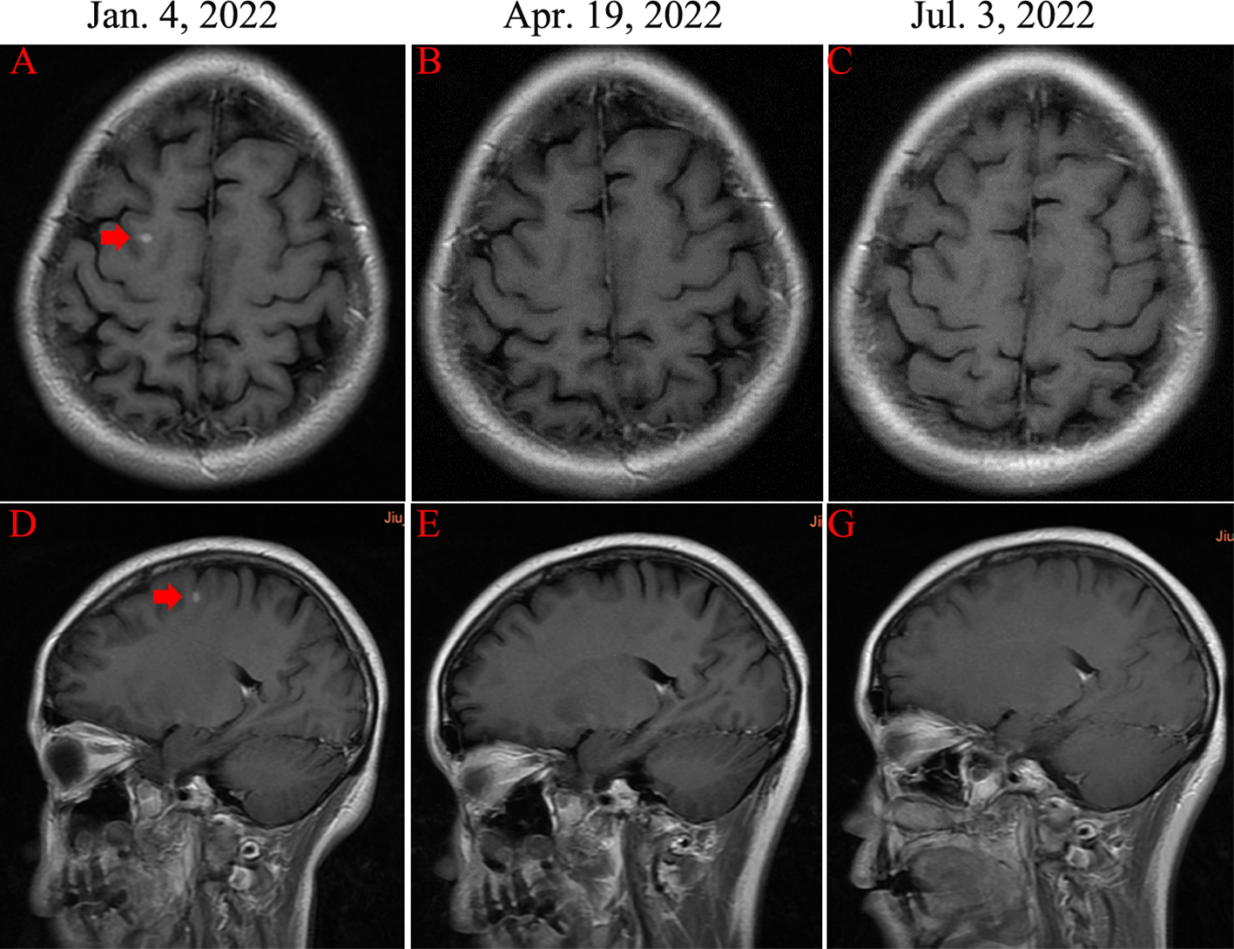
Magnetic resonance imaging (MRI) examinations of right frontal lobe metastasis (red arrow). **(A, D)** The presence of solid brain metastasis (diameter of 1 cm). **(B, E)** and **(C, G)** The disappearance of brain metastasis.

**Figure 4:**
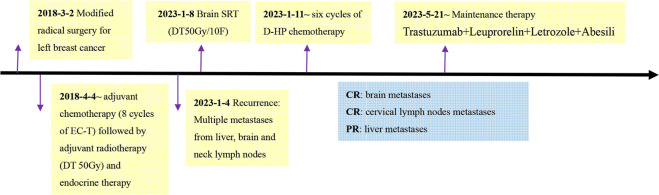
Detailed time course of the patient's clinical course and therapeutic regimens.

After 3 cycles of treatment, the clinical efficacy showed partial remission (PR). After 6 cycles of treatments, the patient achieved complete remission (CR) of cervical metastatic lymph nodes and brain metastasis (**Figure 3**). For liver metastases, the clinical efficacy showed partial remission (PR). Since May 21, 2023, the patient began to receive maintenance therapy, which included trastuzumab, leuprorelin, letrozole, and Abemaciclib (an inhibitor of CDK4/6). Currently, the patient is receiving regular maintenance therapy without any clear discomfort. In December 2023, the latest efficacy was evaluated, and the hepatic metastatic lesions almost disappeared, indicating nCR (**Figure 2**). She has achieved progression-free survival (PFS) of over 12 months and the overall survival of 69 months up to now (**Figure 4**). The patient still undergoes close follow-up and receives regular examination every 6 months.

## Discussion

Previously, it was believed that breast cancer was a static disease in terms of pathology. However, in-depth molecular studies have found that breast cancer should be considered as a spatially and temporally dynamic disease, i.e. that the heterogeneities exist in different sites between primary tumors and metastases, and different phases between initial diagnosis and recurrence (7). The phenomenon of heterogeneity between primary and metastatic lesions has attracted increasing attention from clinicians in the field of breast cancer. Timmer M et al. compared the status of ER, PR, and HER2 in primary tumors with brain metastases in 24 patients with breast cancer. They found that 25-37.5% of patients exhibited discordant receptor status between the primary tumor and brain lesions (4). Nishimura R et al. reported the changes in ER, PR, Ki-67, and HER2 status between primary and recurrent lesions in 97 patients with breast cancer. Following relapse, ER and PR decreased while Ki-67 increased. Subtype changes occurred in 24.7% of the patients (5). Lin M et al. compared the changes in HER2 status between primary tumors and paired recurrent/metastatic lesions in 1299 patients with breast cancer. The incidence of discordance was 28.5% (370/1299), indicating the conversion of primary-to-metastatic HER2 status (3). The results indicated that the heterogeneity between primary and metastatic lesions is an important clinical issue that cannot be ignored.

The underlying mechanisms have been partly ascribed to clonal evolutions, which contribute to genetic heterogeneity during malignant progression. Sprouffske K et al. faithfully mimicked the clonal evolution of metastasis process in the patient-derived tumor xenografts (PDX) from breast cancer. During the process of clonal selection, some subclones remain stable, some expand, and others vanish over time. Furthermore, clonal evolution leads to genetic heterogeneity and different clinical outcomes (8). A prospective analysis of circulating tumor DNA (ctDNA) was performed by next-generation sequencing (NGS) in 37 patients with HR-positive, HER2-negative breast cancer who were classified as secondary resistance. The occurrences of new mutations were 0% (0/9) for the chemotherapy group, 42.1% (8/19) for the CDK4/6 inhibitors plus endocrine treatment (CE group), and 36.3% (4/11) for the chemotherapy followed by CE group (p=0.024). The results strongly indicated that chemotherapy or CDK4/6 inhibitors may influence the change of clonal evolution among breast cancer patients (9). Liquid biopsy by Kujala J et al. detected 56.2 ± 7.2% of somatic variants present both in the matched primary tumor and metastatic sites from breast cancer patients. Apparently, liquid biopsy may identify novel driver variants and therapeutic targets absent from the tumor tissue DNA (10). In the clinical setting, ctDNA may be an ideal surrogate for longitudinal monitoring of genetic heterogeneity from breast cancer patients who can’t obtain tissue specimen (7). Another potential explanation may be attributed to that a second local lesion (*in situ* or invasive) could have developed concomitantly with the primary DCIS or in the period between the primary tumor and recurrence, which could be a plausible explanation for the luminal metastases. Thus, it is necessary and important to follow-up the patients with breast cancer.

According to the studies, all the insistences occurred in only one or two of three receptors (ER, PR, and HER2 receptors) in breast cancer patients at the stages of initial diagnosis and recurrence & metastasis (3–5). However, the transition from triple-negative to ER, PR, and HER2 positive status was rarely observed. In our report, we present a case of a female patient newly diagnosed with triple-negative breast cancer, who experienced multiple metastases to the liver, brain, and cervical lymph nodes 57 months after the operation. A re-biopsy of the liver metastases revealed a conversion into ER, PR, and HER2 positive status. In this case, the liver was the optimal site available for re-biopsy. As a result, the patient received accurate detection and precision medicine. Encouragingly, the patient achieved nCR for all the metastases after systematic treatments. This re-confirmed the correct diagnosis after disease relapse and metastasis. In contrast, re-biopsy from brain metastasis is not easily available for breast cancer patients. In this clinical setting, liquid biopsy of ctDNA [including cerebrospinal fluid (CSF)] may be an alternative option for these types of patients (11).

## Conclusions

The discrepancy in the biomarkers of ER, PR, and HER2 status presents a significant clinical challenge in patients with breast cancer, particularly between primary tumors and metastatic lesions. Simultaneous conversion of all three biomarkers is a rare occurrence during the disease progression of breast cancer. This study presents a case of a female patient with initial triple-negative breast cancer who transformed to ER, PR, and HER2-positive status after recurrence and metastasis. Consequently, she underwent systematic treatment, including chemotherapy, targeted therapy, cranial radiotherapy, and maintenance treatment. The patient achieved a promising complete response (nCR) for over 12 months up to now. The final results of CLEOPATRA study reported that mOS and 8-year OS rates were significantly higher in the D-HP group than that in was D-H (docetaxel-trastuzumab) group (*mOS*: 57.1 *vs*. 40.8 months; *8-year OS rates*: 37% *vs*. 23%) (12). Therefore, we have reasons to believe that the patient is likely to have an excellent outcome. This report highlights the necessity and importance of secondary biopsy in patients with breast cancer, which could significantly facilitate precise diagnosis and treatment.

## Data availability statement

The original contributions presented in the study are included in the article/supplementary material. Further inquiries can be directed to the corresponding authors.

## Ethics statement

The studies involving humans were approved by Ethic Committee of Jiujiang University Affiliated Hospital. The studies were conducted in accordance with the local legislation and institutional requirements. The participants provided their written informed consent to participate in this study. Written informed consent was obtained from the individual(s) for the publication of any potentially identifiable images or data included in this article.

## Author contributions

JH: Investigation, Methodology, Writing – original draft. LL: Data curation, Formal analysis, Methodology, Writing – original draft. JD: Conceptualization, Software, Writing – review & editing.

## References

[1] Tao X Li T Gandomkar Z Brennan PC Reed WM . Incidence, mortality, survival, and disease burden of breast cancer in China compared to other developed countries. Asia Pac J Clin Oncol. (2023) 19:645–54. doi: 10.1111/ajco.13958 37026375

[2] Loibl S Poortmans P Morrow M Denkert C Curigliano G . Breast cancer. Lancet. (2021) 397:1750–69. doi: 10.1016/S0140-6736(20)32381-3 33812473

[3] Lin M Luo T Jin Y Zhong X Zheng D Zeng C . Her2-low heterogeneity between primary and paired recurrent/metastatic breast cancer: implications in treatment and prognosis. Cancer. (2023) 130:851–62. doi: 10.1002/cncr.35101 37933913

[4] Timmer M Werner JM Rohn G Ortmann M Blau T Cramer C . Discordance and conversion rates of progesterone-, estrogen-, and her2/neu-receptor status in primary breast cancer and brain metastasis mainly triggered by hormone therapy. Anticancer Res. (2017) 37:4859–65. doi: 10.21873/anticanres.11894 28870906

[5] Nishimura R Osako T Okumura Y Tashima R Toyozumi Y Arima N . Changes in the er, pgr, her2, p53 and ki-67 biological markers between primary and recurrent breast cancer: discordance rates and prognosis. World J Surg Oncol. (2011) 9:131. doi: 10.1186/1477-7819-9-131 22004841 PMC3214885

[6] Pellas U Bauer A Baros IV Fattorini C Tot T . Her2-low metastases of her2-negative primary tumors: a single institution analysis of intertumoral and internodal heterogeneity in node-positive breast cancer. Front Oncol. (2023) 13:1167567. doi: 10.3389/fonc.2023.1167567 37483511 PMC10362429

[7] Kavan S Kruse TA Vogsen M Hildebrandt MG Thomassen M . Heterogeneity and tumor evolution reflected in liquid biopsy in metastatic breast cancer patients: a review. Cancer Metastasis Rev. (2022) 41:433–46. doi: 10.1007/s10555-022-10023-9 35286542

[8] Sprouffske K Kerr G Li C Prahallad A Rebmann R Waehle V . Genetic heterogeneity and clonal evolution during metastasis in breast cancer patient-derived tumor xenograft models. Comput Struct Biotechnol J. (2020) 18:323–31. doi: 10.1016/j.csbj.2020.01.008 PMC7026725 32099592

[9] Decker T Bichler M Birtel A Fischer G Geiger K Gaenger S . Clonal evolution in patients with hormone receptor positive, her-2 negative breast cancer treated with chemotherapy or cdk4/6 inhibitors. Oncol Res Treat. (2022) 45:248–53. doi: 10.1159/000523758 35220309

[10] Kujala J Hartikainen JM Tengstrom M Sironen R Auvinen P Kosma VM . Circulating cell-free dna reflects the clonal evolution of breast cancer tumors. Cancers (Basel). (2022) 14:1332. doi: 10.3390/cancers14051332 PMC8909912 35267640

[11] Fitzpatrick A Iravani M Mills A Vicente D Alaguthurai T Roxanis I . Genomic profiling and pre-clinical modelling of breast cancer leptomeningeal metastasis reveals acquisition of a lobular-like phenotype. Nat Commun. (2023) 14:7408. doi: 10.1038/s41467-023-43242-x 37973922 PMC10654396

[12] Swain S Miles D Kim S Im Y Im S Semiglazov V . Pertuzumab, trastuzumab, and docetaxel for HER2-positive metastatic breast cancer (CLEOPATRA): end-of-study results from a double-blind, randomised, placebo-controlled, phase 3 study. Lancet Oncol. (2020) 21:519–30. doi: 10.1016/S1470-2045(19)30863-0 32171426

